# Dual-layer spectral detector computed tomography multiparameter machine learning model for prediction of lymph node metastases in esophageal squamous cell carcinoma

**DOI:** 10.1186/s40644-026-00993-2

**Published:** 2026-01-29

**Authors:** Junjie Zhang, Ligang Hao, Peiyi Ma, Qiuxu Zhang, Linyi Jia, Fengxiao Gao

**Affiliations:** 1 Department of Computed Tomography and Magnetic Resonance, Xing Tai People’s Hospital, 818 Xiangdu Road, Xing Tai, Hebei 054000 China; 2 Department of Thoracic Surgery, Xing Tai People’s Hospital, Xing Tai, He Bei 054000 China; 3 Department of Laboratory Medicine, QingHe Hospital of Traditional Chinese Medicine, QingHe, Xing Tai, He Bei 054800 China

**Keywords:** Esophageal squamous cell carcinoma (ESCC), Lymph node metastasis (LNM), Dual-layer spectral detector CT (SDCT), Nomogram

## Abstract

**Objective:**

Accurate assessment of lymph node metastasis (LNM) is essential for the staging, treatment, and prognosis of esophageal squamous cell carcinoma (ESCC). This study investigates the potential of dual-layer spectral detector computed tomography (SDCT) quantitative parameters in predicting LNM in ESCC.

**Method:**

The study included 158 patients with pathologically confirmed ESCC, comprising 92 patients without LNM and 66 patients with LNM. The chi-square test or Fisher’s exact test was utilized to analyze the basic clinical data and lymph node morphological features of the patients. To evaluate the differences in various SDCT quantitative parameters between the LNM and non-LNM groups, the Mann-Whitney U-test and independent sample t-test were applied. Patients were randomly assigned to training and test groups in a 7:3 ratio. The area under the receiver operating characteristic (ROC) curve (AUC) was used to assess the machine learning model. Furthermore, decision curve analysis (DCA) was performed to evaluate the model’s diagnostic efficacy, leading to the development of a nomogram.

**Results:**

Both univariate and LASSO analyses identified LAD, SAD, CT_V−40keV_, NIC_V_, ED, ECV_V_, and Nct as significant predictors of LNM in EC. The logistic regression model demonstrated superior performance, achieving ROC-AUC values of 0.885 and 0.827 in the training and test cohorts, respectively. The Brier scores for the combined model were 0.135 and 0.172 in the training and test cohorts, respectively.

**Conclusion:**

The logistic regression model, which integrates SDCT quantitative parameters and lymph node morphological features, exhibited substantial diagnostic value for assessing LNM in EC. It demonstrated excellent diagnostic efficacy and provides a non-invasive evaluation method for clinical practice.

## Introduction

In 2022, esophageal cancer (EC) was identified as the 11th most commonly diagnosed cancer globally, with 511,000 new cases, and it was the seventh leading cause of cancer-related mortality, accounting for 445,000 deaths [1]. In China, ESCC was more common in patients with EC. And patients with ESCC are often diagnosed at advanced stages, rendering them ineligible for surgical intervention and resulting in a poor prognosis, with 5-year survival rates ranging from 20% to 30% [2]. LNM is a critical factor in determining the prognosis and survival outcomes of patients [3]. In its eighth edition, the AJCC International Staging Standard for EC introduced a clinical staging system that incorporates preoperative imaging and includes the enumeration of LNM in the postoperative stage [4, 5]. Although pathological examination remains the gold standard for diagnosing LNM, lymph node biopsy is invasive and associated with a significant risk of complications [6]. Therefore, accurate assessment of lymph node status through noninvasive imaging techniques is crucial for making informed treatment decisions and providing precise prognostic evaluations.

CT scans are widely utilized as a noninvasive imaging modality for acquiring preoperative and postoperative tumor data to evaluate lymph node status in patients with EC [7]. Nonetheless, when relying solely on morphological criteria, such as short-axis diameter and shape, which are interpreted by clinicians with varying levels of diagnostic expertise, conventional CT scans demonstrate inadequate accuracy in detecting LNM. The diagnostic precision of traditional CT scans is limited, with sensitivity ranging from 37.3% to 67.2% and specificity from 63.9% to 96.4% [8]. Furthermore, these criteria result in particularly low diagnostic accuracy for lymph nodes of normal size, thereby complicating the assessment of lymph node status using traditional CT scans.

SDCT utilizes the rapid alternation between high- and low-energy X-rays to generate multiple virtual monochromatic images, thereby facilitating material decomposition. This technology aids in diagnosing various diseases and in nodal staging, including conditions such as thyroid carcinoma and lung cancer [9–12]. Empirical evidence indicates that SDCT effectively distinguishes benign tumors from malignant ones and predicts LNM [13–15]. Previous research on SDCT’s capacity to predict LNM has primarily concentrated on assessing whether lymph nodes associated with different malignant tumors are metastatic by extracting quantitative data about these nodes. However, establishing a direct correlation between SDCT findings and pathological outcomes for the selection and evaluation of lymph nodes in clinical practice remains challenging, frequently leading to inconsistencies in analytical results. Consequently, there is growing interest in predicting LNM by analyzing the key characteristics of primary tumors rather than lymph nodes, a method that has proven effective in certain studies on malignant tumor lymph nodes. Notably, there is a paucity of research focused on predicting LNM by examining the primary cancer sites of ESCC using SDCT [16–18]. Few comprehensive evaluation systems currently integrate diverse combinations of lesion morphology and spectral quantitative parameters in ESCC. Consequently, the aim of this study was to examine the significance of SDCT quantitative parameters and their derivatives, in conjunction with lesion morphology, for predicting LNM in ESCC. Furthermore, we developed a predictive model for LNM to aid in identifying the most appropriate treatment strategy.

## Materials and methods

The research received approval from the Institutional Review Board of Xing Tai People’s Hospital (Xing Tai People’s Hospital Ethics Committee, reference number: 2022KS017, date: June 27, 2022), with individual consent being waived for this retrospective analysis. This study adhered to the principles of the Declaration of Helsinki, revised in 2013.

### Patient selection

This study adheres to the STROBE guidelines and involves a retrospective analysis of data from patients admitted to our hospital between January 2021 and December 2024 who underwent radical surgery and systematic lymph node dissection. Eligibility criteria for inclusion in the study were as follows: (1) patients must have undergone a SDCT chest-enhanced scan at our institution; (2) a diagnosis of squamous cell carcinoma confirmed by surgical pathology; and (3) availability of comprehensive clinical examination data, including clinical records and spectral enhanced CT imaging data, obtained within two weeks prior to surgery. Exclusion criteria included: (1) a diagnosis of cervical esophageal cancer or gastroesophageal junction carcinoma; (2) receipt of preoperative neoadjuvant therapy or other anti-tumor treatments; (3) primary tumors that were non-palpable or too small to be measured; and (4) the presence of other types of cancer. Ultimately, 158 patients were included in the study. Based on pathological evaluation of the lymph nodes, participants were classified into the non-LNM group (*n* = 92) and the LNM group (*n* = 66).

### SDCT image acquisition

The patients underwent scanning using a SDCT system (IQon; Philips Healthcare, Best, The Netherlands) following a standardized protocol for chest enhancement. All participants were positioned in a supine orientation, with the scan range extending from the thoracic inlet to the costophrenic angle. The scanning parameters were configured as follows: tube voltage was set at 120 kVp, with three-dimensional tube current modulation; the matrix size was 512 × 512; collimator width was 64 × 0.625 mm; the scan field of view measured 372 mm; spacing was 0.90 mm; and the rotation time was 0.50 s. The scanning layer thickness was 5 mm, while the reconstruction layer was 1 mm. A contrast agent (iodofol: 1.0–1.5 mL/kg, iodine concentration: 350 mg/mL) was administered via the antecubital vein at a rate of 3.0 mL/s, followed by a 20 mL injection of normal saline at the same rate. Dual-phase enhanced scans were performed 30 s post-contrast agent injection for the arterial phase and 90 s for the venous phase. The reconstructed spectral-based images (SBIs) were subsequently transferred to a dedicated workstation (IntelliSpace Portal V10, Philips Healthcare) for spectral parameter analysis.

### Acquisition and analysis of SDCT quantitative parameters

For the purpose of analysis and processing, all images were uploaded to the IntelliSpace Portal (Philips Healthcare, Best, The Netherlands) utilizing the Spectral CT Viewer provided with the workstation. Image analysis was performed by radiologist Ma Peiyi and overseen by senior radiologist Zhang Junjie. The assessment employed mediastinal window images (width: 400 HU; level: 40 HU), which were adjusted as necessary to optimize visualization of the tumor tissue. Following the identification of lesions during film reading, ROI was manually delineated at three consecutive levels, including the level containing the largest lesion and the adjacent levels above and below it. To ensure accurate measurements, regions containing blood vessels and necrosis were excluded. The mean of three measurements was calculated for each patient to derive the final analytical data. To standardize the SDCT quantitative parameters, the ROI of the aorta at comparable layers was determined using the same methodology. This process yielded a comprehensive set of parameters.

In our study, we recorded the following values: the CT values of the lesions at VNC, arterial phase, and venous phase (40 and 70 keV), denoted as CT_VNC_, CT_A−40keV_, CT_A−70keV_, CT_V−40keV_, and CT_V−70keV_, respectively. Concurrently, we calculated the attenuation coefficient (λ) for both the arterial and venous phases using the following formulas: λ_A_=(CT_A−40keV_- CT_A−70keV_)/ (70 − 40), λ_V_=(CT_V−40keV_- CT_V−70keV_)/ (70 − 40). The iodine concentration (IC, mg/mL) of the lesions and the aorta during the arterial and venous phases were recorded as IC _A−tumor_, IC _A−aorta_, IC _V−tumor_, and IC _V−aorta_, respectively. To account for inter-patient hemodynamic variability, the IC values were normalized to the aorta. The normalized iodine concentration (NIC) was calculated using the following formulas: NIC_A_ = IC _A−tumor_/ IC _A−aorta_, NIC_V_ = IC _V−tumor_/ IC _V−aorta_. The additional spectral qualitative parameters analyzed included the Zeff, ED, AEF and ECV fraction. The evaluation of Zeff and ED was conducted using images from the venous phase. In contrast, AEF and ECV assessments were based on the iodine density within the lesion. The ECV measurements of the lesions during the arterial and venous phases were denoted as ECV_A_ and ECV_V_, respectively.

The greatest tumor thickness and length, considered as potential predictive features, were measured on the maximum axial and sagittal images, respectively. Simultaneously, we performed an evaluation of Nct using CT images, which relies on observing the esophagus in CT mixed energy images. Regional lymph nodes with a shortest diameter of ≥ 10 mm and/or significant uneven enhancement are considered Nct positive, otherwise Nct negative. To assess interobserver repeatability and variability, a random selection of 50% of the participants (79 out of 158) was made, and another radiologist (Jia Linyi, with 20 years of experience), independently repeated the initial measurement procedure.

### Statistical analysis and model establishment

All statistical analyses were conducted using Python version 3.7. Baseline characteristics were evaluated employing the Wilcoxon rank-sum test for continuous variables and either the χ2 test or Fisher’s exact test for categorical variables. The Spearman correlation coefficient was utilized to assess the relationships between features. Independent CT features associated with LNM were identified through both univariable and the LASSO algorithm was employed to select relevant independent features. All statistical tests were two-tailed, with a P value of less than 0.05 considered indicative of statistical significance. Risk factors were identified using optimization algorithms, followed by the construction of a nomogram. The model’s performance was assessed on both the training and test datasets using the area under the AUC and the precision-recall curve, with ORs calculated for each selected variable. Additionally, DCA was employed to evaluate the model’s clinical utility by calculating net benefits at various threshold probabilities.

## Results

### Statistical analysis of the basic clinical data

This research involved 158 patients with ESCC who had radical surgery and systematic lymph node dissection. LNM was detected in 66 patients (41.8%, 43 males and 23 females), while 92 patients (58.2%, 60 males and 32 females) showed no LNM. Table 1 presents the analysis of clinical and SDCT quantitative parameters for the 158 patients included. There were no significant differences in gender, age, and tumor location between the groups (*P* > 0.05).

**Table 1:** Clinical characteristics and radiomics features of the patients

Characters	LNM-	LNM+	*p*
Age, Mean	66.500	67.000	0.615
Gender			1.000
Female	60	43	
Male	32	23	
Nct			0.041
Negative	80	48	
Positive	12	18	
LAD, Mean (SD)	42.02 (14.85)	48.11 (16.51)	0.019
SAD Mean (SD)	12.15 (9.38;15.20)	13.35 (11.50;16.20)	0.030
CT_VNC_, Mean (SD)	40.94 (6.50)	41.57 (5.76)	0.524
CT_A−40keV_,Median (95%CI)	117.40 (93.73;138.00)	116.95 (102.95;140.48)	0.661
CT_A−70keV_, Mean(SD)	63.85 (12.31)	65.06 (9.36)	0.484
λ_A_, Median(95%CI)	1.77(1.26;2.28)	1.78 (1.43;2.35)	0.698
IC _A−tumor_, Median (95%CI)	0.90 (0.63;1.17)	0.88 (0.73;1.11)	0.839
IC _A−aorta_, Mean(SD)	10.36 (2.16)	10.59 (1.96)	0.483
NIC_A_, Median(95%CI)	0.09 (0.07;0.10)	0.090(0.061;0.11)	0.735
CT_V−40keV_, Mean(SD)	136.51(30.93)	147.66(26.60)	0.016
CT_V−70keV_, Mean(SD)	69.23 (11.51)	74.09 (9.61)	0.005
λ_V_, Mean(SD)	2.24 (0.71)	2.484 (0.60)	0.019
IC _V−tumor_, Mean(SD)	1.10 (0.36)	1.24 (0.30)	0.009
IC _V−aorta_, Mean(SD)	3.05 (0.61)	3.14 (0.63)	0.386
NIC_V_, Median(95%CI)	0.38(0.30;0.43)	0.51 (0.41;0.68)	< 0.001
Zeff, Median(95%CI)	7.94(7.81;8.06)	8.02(7.93;8.13)	0.008
ED, Median(95%CI)	104.20(103.78;104.60)	104.45(104.10;104.90)	0.001
AEF, Median(95%CI)	23.45(19.60;27.64)	23.45(20.80;26.68)	0.889
ECV_A_, Median(95%CI)	5.30(4.28;5.83)	5.10(4.23;5.89)	0.715
ECV_V_, Mean(SD)	21.40 (5.13)	23.19 (4.64)	0.023

LAD: tumor length measured by CT as long-axsis diameter, SAD: tumor thickness measured by CT as short-axis diameter. The CT values of the lesions at virtual non-contrast (VNC), arterial phase, and venous phase (40 and 70 keV), denoted as CT_VNC_, CT_A-40keV_, CT_A-70keV_, CT_V-40keV_, and CT_V-70keV_, respectively. Concurrently, we calculated the attenuation coefficient (λ) for both the arterial and venous phases using the following formulas: λ_A_=(CT_A-40keV_- CT_A-70keV_)/ (70-40), λ_V_=(CT_V-40keV_- CT_V-70keV_)/ (70-40). The iodine concentration (IC, mg/mL) of the lesions and the aorta during the arterial and venous phases were recorded as IC _A-tumor_, IC _A-aorta_, IC _V-tumor_, and IC _V-aorta_, respectively. The normalized iodine concentration (NIC) was calculated using the following formulas: NIC_A_ = IC _A-tumor_/ IC _A-aorta_, NIC_V_ = IC _V-tumor_/ IC _V-aorta_. The additional spectral qualitative parameters analyzed included the effective atomic number (Zeff), electron density (ED), arterial enhancement fraction (AEF), and extracellular volume (ECV) fraction. Nct: lymph node status

### Comparison of the SDCT quantitative parameters between the non-LNM and LNM groups

Figures 6 and 7 provide schematic representations of the quantitative parameters associated with the primary lesions and the delineation of ROI for the two groups, respectively. The quantitative parameters of SDCT and their derived metrics were obtained through consistent ROI delineation and normalization processes. In the statistical analysis, encompassing both quantitative and derived quantitative measures, were evaluated. Table 1 summarizes the results of the analysis concerning variations in quantitative parameters of the primary lesions across both groups. Notably, 10 quantitative parameters, including LAD, SAD, CT_V−40keV_, CT_V−70keV_, λ_V_, IC_V−tumor_, NIC_V_, Zeff, ED, and ECV_V_, were significantly elevated in the LNM group compared to the non-LNM group. Conversely, no significant differences were observed between the groups for other quantitative measures such as CT_VNC_, CT_A−40keV_, and ECV_A_, with P-values exceeding 0.05.

### LASSO analyses of LNM

**Fig. 1:**
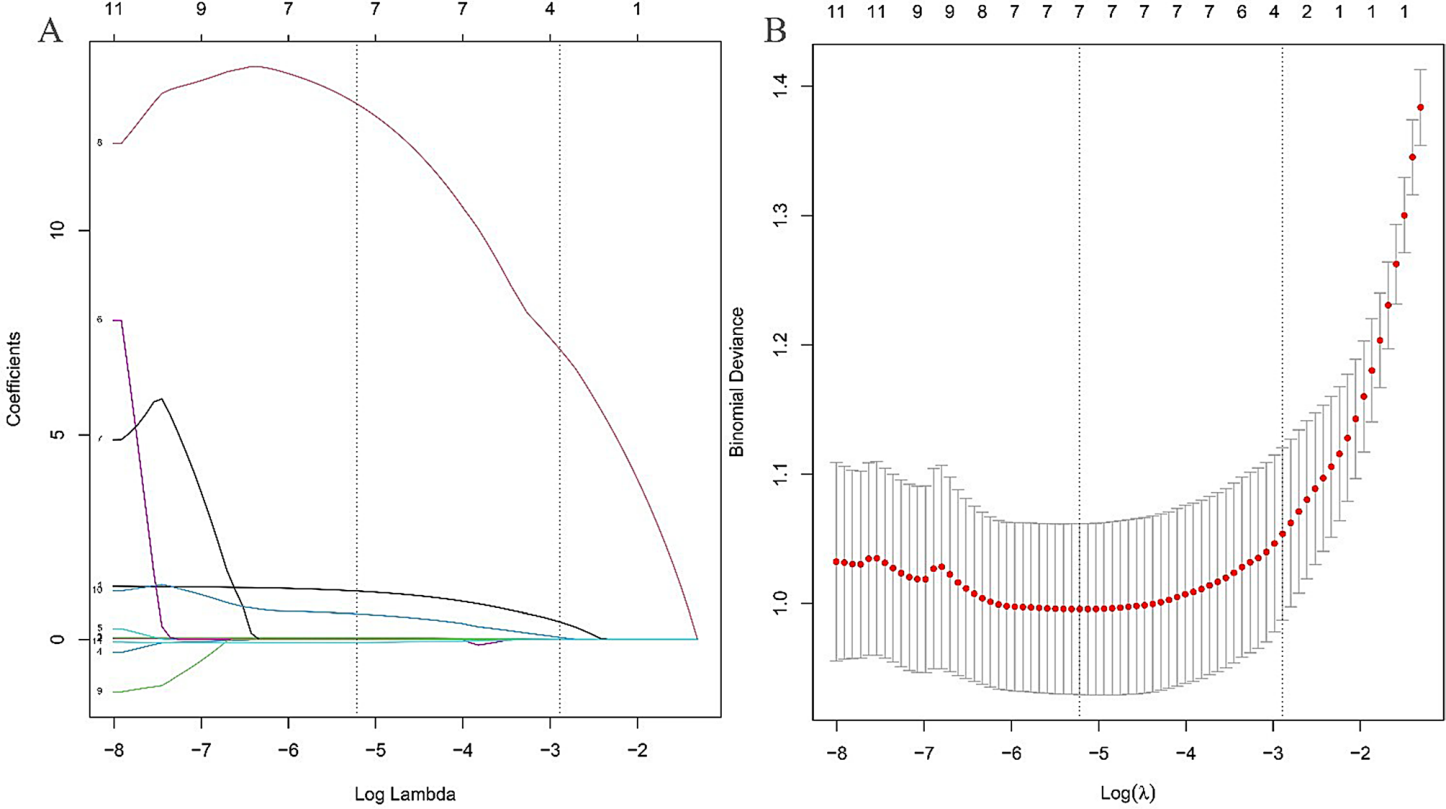
features selection with LASSO. **A**: x-axis represents log (λ), and the numbers above the x-axis represent the average number of predictive variables. The red dot represents the average deviation value of each model with a given λ, while the vertical bar of the red dot represents the upper and lower limit values of the deviation. The vertical dotted line represents the log (λ) value corresponding to the best λ value; the selection standard is the minimum standard. By adjusting different parameters (λ), the binomial deviation of the model is minimized, and the feature datasets with the best performance are selected. **B**: Plots the coeffificients of the log (λ) function. The dotted line represented the λ value the minimum standard and the smallest. Select the coeffificient that is not 0 here as the coeffificient of the last reserved feature. After screening out the redundant features by LASSO, the seven most robust features LAD, SAD, CT_V_-40kev, NIC_V_, ED, ECV_V_, and Nct) were retained, with λ = 0.005

Figure 1 presents the outcomes of LASSO analyses concerning SDCT quantitative parameters predictive of LNM. After screening out the redundant features by LASSO, the seven most robust features were identified LAD, SAD, CT_V−40kev_, NIC_V_, ED, ECV_V_, and Nct as significant determinants of LNM(Fig. 1).

### Model establishment and evaluation

**Fig. 2:**
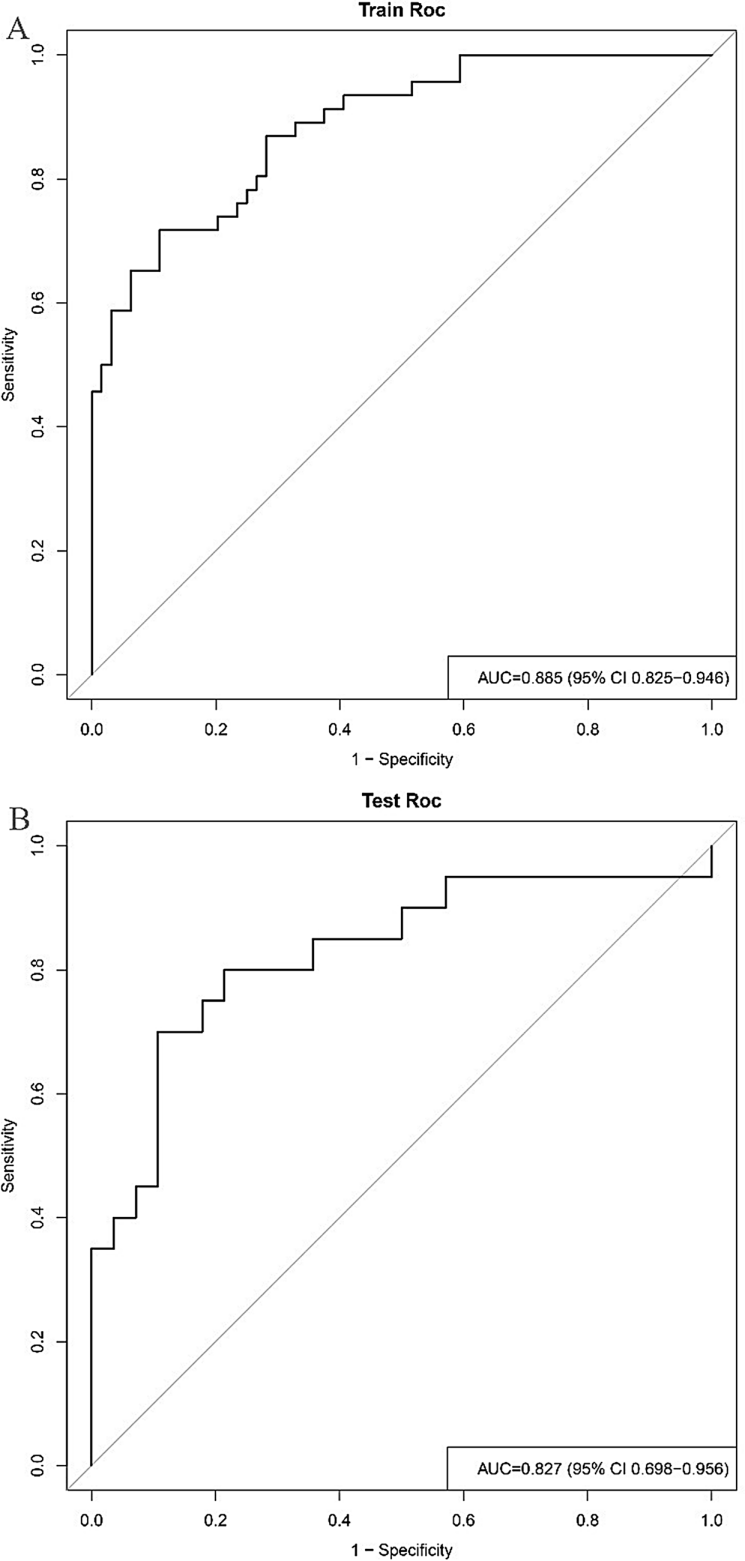
Receiver operating characteristics (ROC) curves of the LR learning in training dataset and testing dataset. The AUCs were 0.885 for the training cohort and 0.827 for the test cohort

**Fig. 3:**
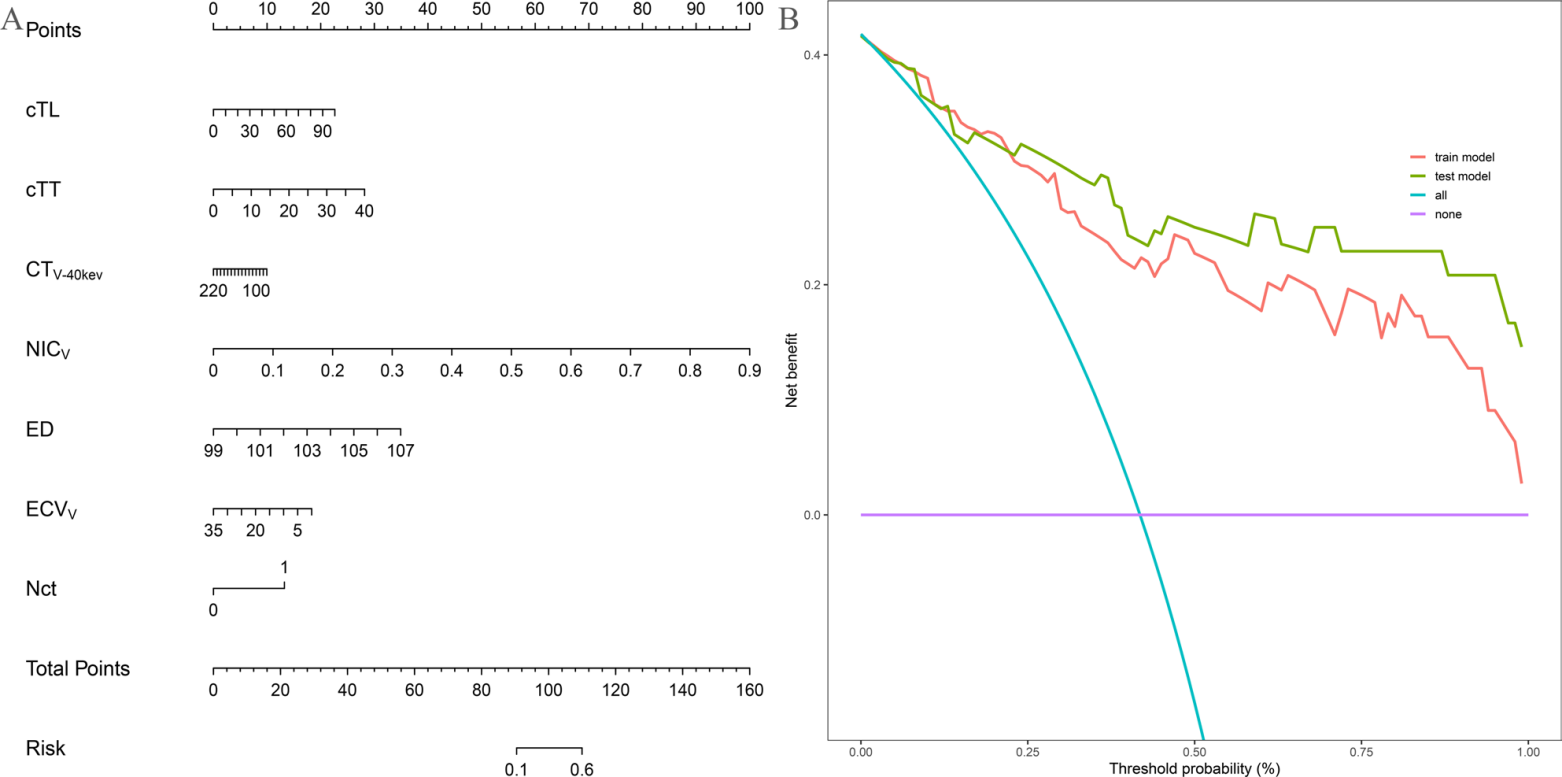
Decision curve analyses and the nomograph for the LR model. **A** nomogram based on logistic regression, **B** DCA curve of established model

**Fig. 4:**
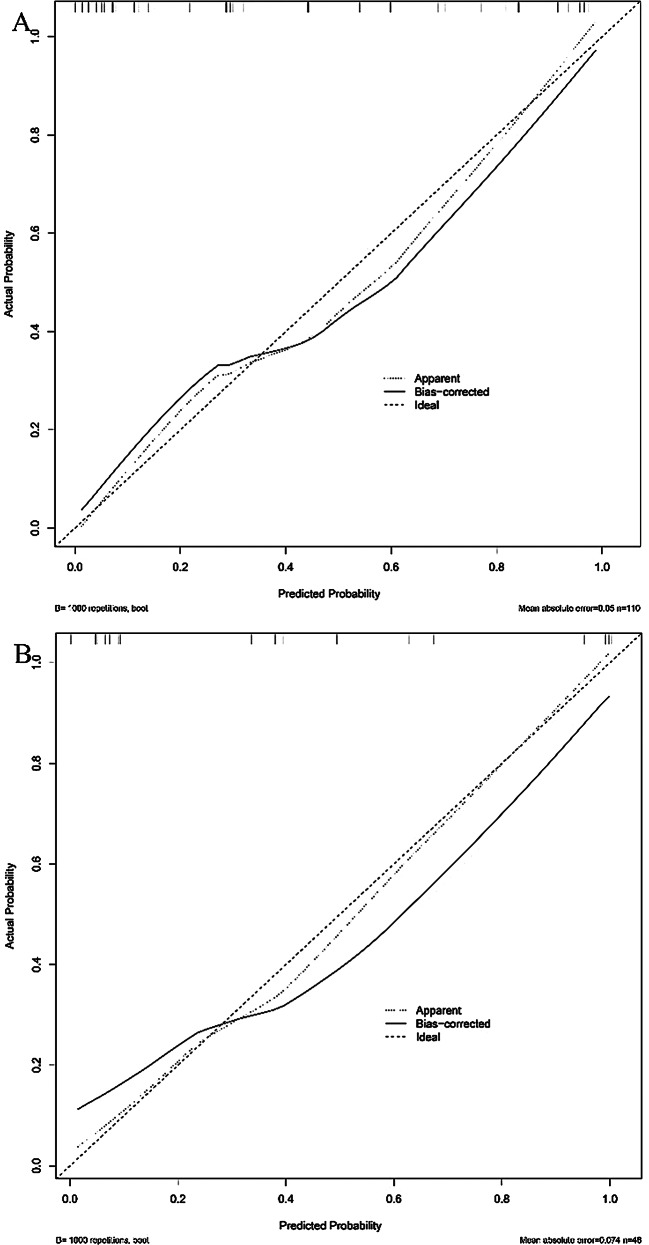
Fitted curves of the LR learning in training dataset and testing dataset. The Brier scores for the prediction model were 0.135 in the training cohort and 0.172 in the test cohort

In this study, a multivariate logistic regression model was employed to identify seven imaging features: LAD, SAD, CT_V−40kev_, NIC_V_, ED, ECV_V_, and Nct. As depicted in Fig. 2, the ROC curve of the predictive model demonstrates that the AUCs were 0.885 for the training cohort and 0.827 for the test cohort. Furthermore, the DCA curves indicated that the prediction model was advantageous for making significant clinical decisions in both the training and test cohorts (Fig. 3B). The Brier scores for the prediction model were 0.135 in the training cohort and 0.172 in the test cohort, as illustrated in the calibration plots in Fig. 4. These seven predictors were subsequently utilized to construct a nomogram model designed to predict LNM in EC, thereby transforming the prediction model into a practical application tool (Fig. 3A). The nomogram points and the risk were caculated based on the nomogram, and the best cut-off value of the risk to predict LNMwas 0.688.

**Fig. 5:**
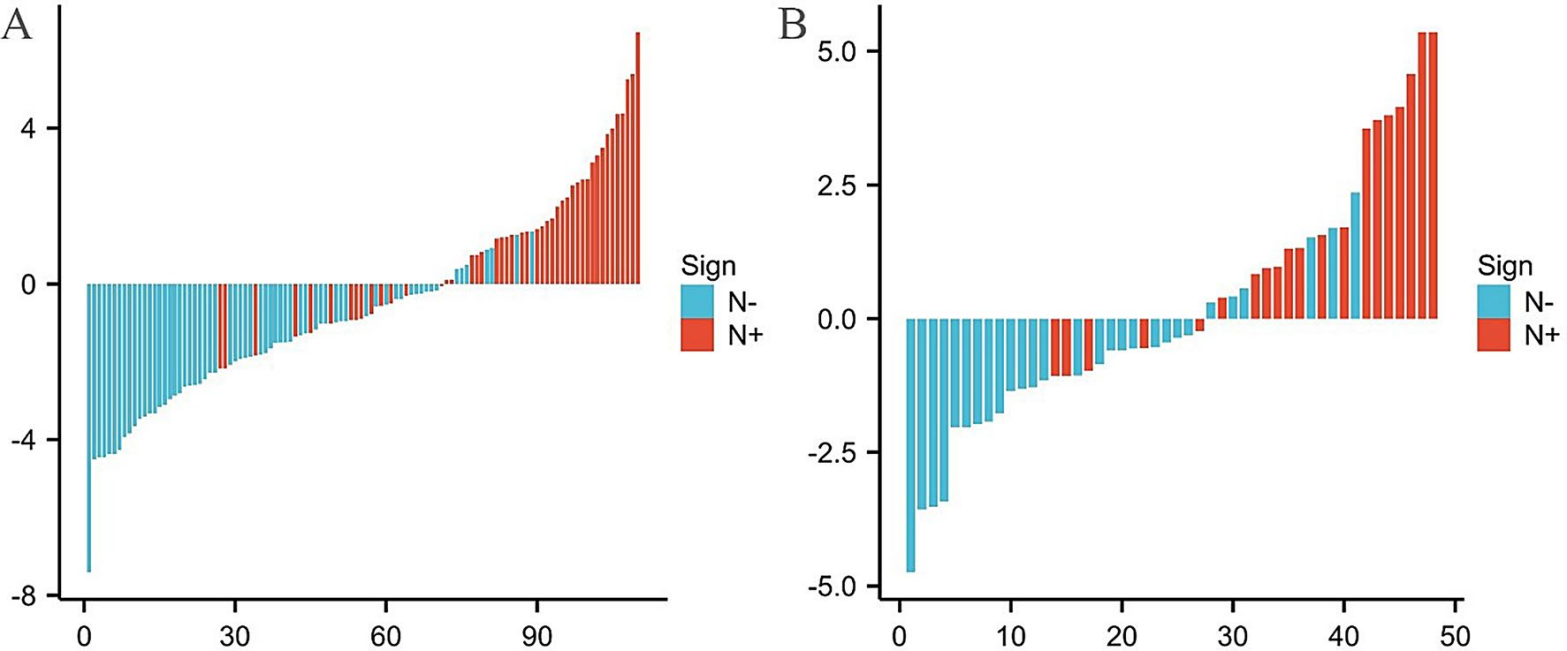
Bar charts of predictive score for each patient in the training cohort (**A**) and testing cohort (**B**). The X-axis represents each patient, each bar represents one patient. Red bars indicate the predictive score for patients with N+, while blue bars indicate the predictive score for patients with N-. Red bars above zero-line or blue bars below the zero-line mean misclassification. The predictive score for each patient was then calculated using the selected features weighted by their respective coefficients in the logistic model, which can be expressed as follows: predictive score = − 67.5 + 0.03*LAD + 0.094*SAD-0.009*CT_V_-40kev + 14.822*NIC_V_+0.582*ED-0.07* ECV_V_+1.765* Nct

The predictive score for each patient was then calculated using the selected features weighted by their respective coefficients in the logistic model, which can be expressed as follows: predictive score = − 67.5 + 0.03*LAD + 0.094*SAD-0.009*CTV-40kev + 14.822*NICV + 0.582*ED-0.07* ECV_V_+1.765* Nct. The predictive core for each patient in the training and testing cohorts is shown in Fig. 5 and the best cut-off value of thepredictive score to predict LNM was 0.744.

**Fig. 6:**
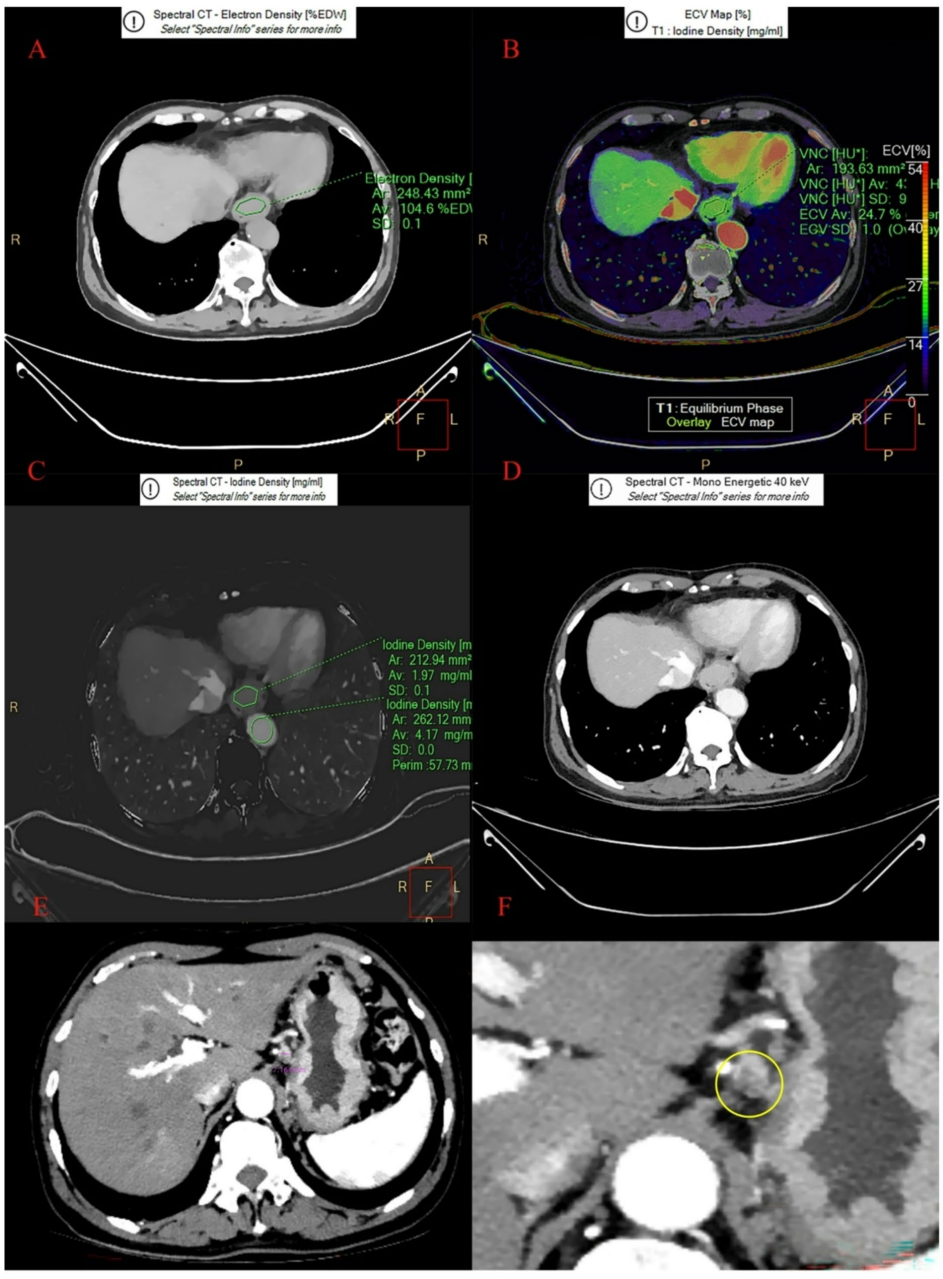
Spectral images in ESCC (female, 72 years old). **A**: ED pseudo-color Images; **B**: ECV pseudo-color images; **C**: Iodine density image in VP. **D**: The 40-keV monochromatic image in the mediastinal window during the VP; **E** and **F**: Nct was negative with the diameter 7.16 mm. LAD 38 mm. SAD 17 mm. CT_V_-40kev: 214.9 HU, NIC_V_: 0.473, ED: 104.6%, ECV_V_: 24.7%. Predictive score=-0.538, nomogram Point = 103.291 and the risk based on the nomogram was 0.381. The best cut-off value of Predictive score and the risk based on the nomogram was 0.744 and 0.688. Based on both score, we predict LNM-, and the pathology confirmed LNM-

**Fig. 7:**
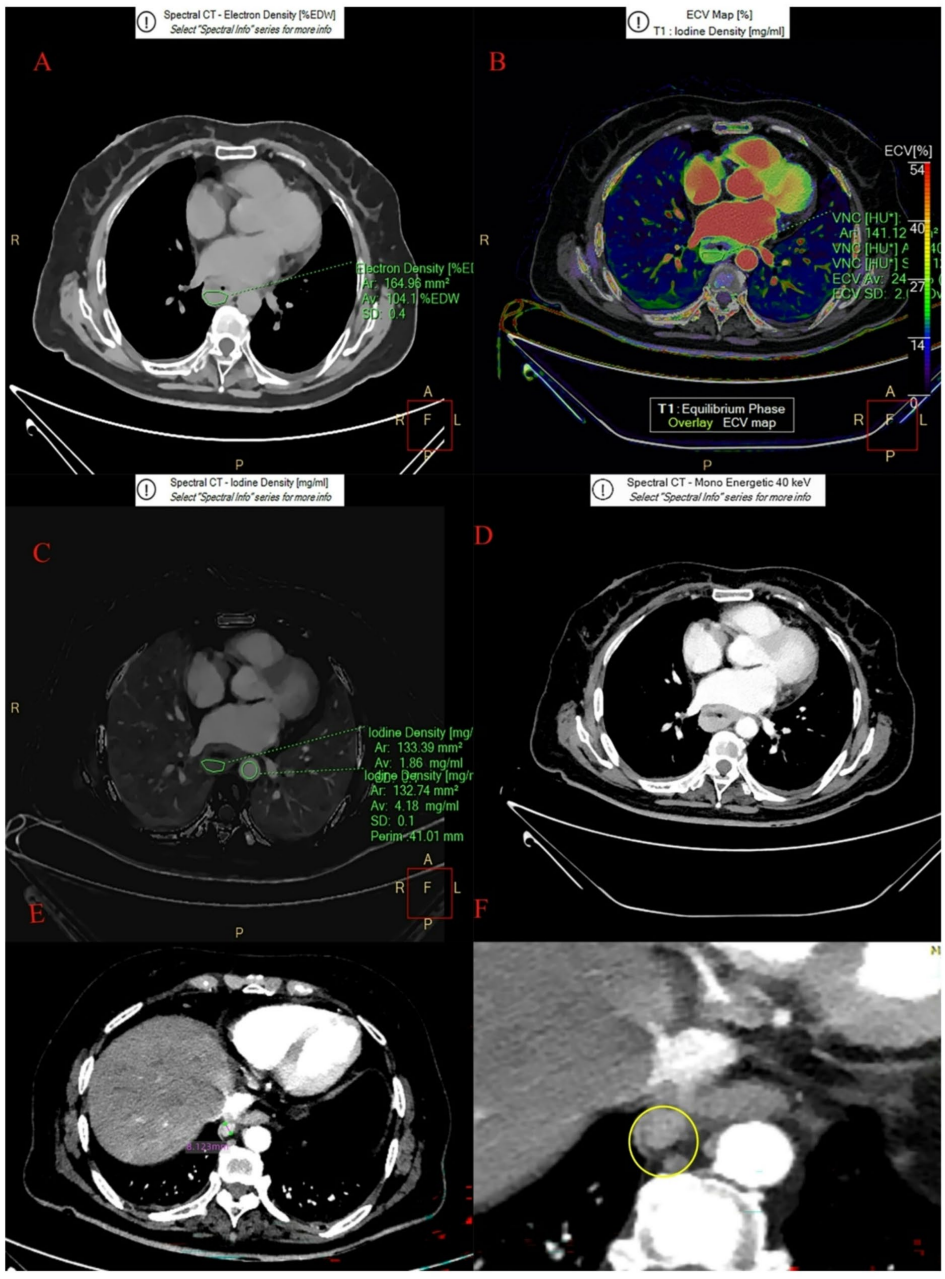
Spectral images in ESCC (female, 69 years old). **A**: ED pseudo-color images; **B**: ECV pseudo-color images; **C**: Iodine density image in VP ; **D**: The 40-keV monochromatic image in the mediastinal window during the VP. **E** and **F**: Nct was negative with the diameter 8.12 mm. LAD 58.5 mm. SAD 17.7 mm. CT_V_-40kev: 195.7Hu, NIC_V_: 0.728, ED: 104%, ECV_V_: 26.9%. Predictive score = 5.361, nomogram Point = 147.523 and the risk based on the nomogram was 0.996. The best cut-off value of Predictive score and the risk based on the nomogram was 0.744 and 0.688. Based on both scores, we predict LNM+, and the pathology confirmed LNM+

Two examples using the LR model to predict LNM as following: Patient 1 (Fig. 6), female, 72 years old. LAD: 38 mm. SAD༚17 mm. Nc: negative. CT_V_-40kev: 214.9 Hu, NIC_V_: 0.473, ED: 104.6%, ECV_V_: 24.7%. Predictive score=-0.538, nomogram Point = 103.291 and the risk based on the nomogram was 0.381. Based on both scores, we predict N-, and the pathology confirmed N-. Patient 2 (Fig. 7), female, 69 years old, LAD༚58.5 mm. SAD༚17.7 mm. Nc: negative. CT_V_-40kev: 195.7Hu, NIC_V_: 0.728, ED: 104%, ECV_V_: 26.9%. Predictive score = 5.361, nomograph Point = 147.523 and the risk based on the nomograph was 0.996. Based on both scores, we predict N+, and the pathology confirmed N+.

## Discussion

ESCC is a highly aggressive malignancy that poses a substantial health burden, particularly in China, where its incidence is notably elevated [19]. This malignancy is distinguished by its rapid progression and poor prognosis, with lymphatic spread serving as a major determinant of patient outcomes [20]. A comprehensive understanding of the biological mechanisms underlying the metastatic process, particularly the role of LNM, is essential for the development of effective treatment strategies. Current diagnostic modalities, although useful, often fail to accurately predict LNM, highlighting the urgent need for novel predictive biomarkers to enhance clinical decision-making and patient management.

In this study, we aimed to explore the potential of spectral CT parameters as predictive markers for LNM in patients with ESCC. Through a retrospective analysis of spectral CT imaging data, integrated with clinical parameters, we sought to develop a logistic regression model for the preoperative prediction of LNM, yielding AUC values of 0.823 and 0.801 in the training and testing sets, respectively. Our findings reveal significant correlations between specific spectral CT parameters and the presence of LNM, thereby offering valuable insights into the utility of advanced imaging techniques for enhancing preoperative assessments in ESCC patients. The implications of these findings may facilitate improved prognostic evaluations and the development of tailored therapeutic strategies in the management of ESCC.

Our research findings suggest that LAD and SAD of ESCC are independent of LNM. In EC, the measurement of tumor thickness on axial CT images and tumor length on sagittal CT images indicates the degree of infiltration and correlates with the tumor’s T stage [20, 21]. An increase in LAD and SAD is associated with a higher probability of LNM. In a study on esophageal carcinoma with invasion depth not reaching the adventitia, multivariate analysis revealed that tumor diameter was an independent risk factor for lymph node metastasis (*p* = 0.005) [22]. This indicates that a larger tumor diameter is associated with a higher likelihood of lymph node metastasis. Another study focusing on the expression of tumor-infiltrating dendritic cells (TIDCs) found that patients with a tumor diameter ≥ 4 cm had significantly lower TIDC density, which was correlated with lymph node metastasis (*P* < 0.05) [23]. This suggests that a larger tumor diameter may increase the risk of lymph node metastasis by influencing the immune microenvironment. In a radiotherapy study for stage III esophageal squamous cell carcinoma, univariable analysis showed that lesion length was associated with overall survival, but it was not retained as an independent prognostic factor in the multivariable analysis; instead, tumor diameter and gross tumor volume were more direct predictive indicators [24]. This suggests that lesion length may be indirectly associated with lymph node metastasis by influencing the overall tumor burden. Our results are consistent with previous studies that have established a relationship between LAD, SAD, and LNM in ESCC. Prior research has demonstrated that each 1 cm increase in tumor length significantly impacts the likelihood of LNM, with an odds ratio (OR) of 3.55 [21]. From a biological perspective, the longitudinal growth within the lymphatic-rich submucosa of the esophagus may be a critical factor contributing to regional LNM [25].

In addition to conventional CT imaging, SDCT can generate a range of energy spectrum parameter images. These images provide essential insights for identifying lesions, accurately assessing their extent, differentiating between benign and malignant lesions, and evaluating the likelihood of recurrence and metastasis [26, 27]. While the SDCT parameters for lymph node assessment have been extensively discussed in the literature, there is a paucity of reports on the spectral CT parameters of LNM in EC. The findings of this study indicate that the CT_V−40keV_ and NIC_V_ are elevated in LNMcompared to non-LNM, corroborating the results of Zhang et al. [28]. and Lu et al. [15]. It may be caused by that tumor cells secrete regulatory factors that initiate microvascular regeneration prior to metastasis [29]. And microvascular regeneration increases a dense vascular presence and the ability to metastasis. So the primary EC with LNM may exhibit a richer blood supply compared to cases without LNM [10]. A substantial number of micro-vessels, characterized by their relative immaturity and high density, contribute to the increased accumulation of contrast agents in EC with LNM. This phenomenon is evidenced by rapid and pronounced contrast enhancement, indicating elevated iodine levels on the iodine density map and larger values in the 40 keV image during the venous phase. In contrast, studies by Li et al. [30]. and Rizzo et al. [31]. reported a significant reduction in IC in these nodes. These findings suggest potential variability among studies concerning the degree of necrosis in metastatic nodes and primary tumors. It may be resulted from that the blood supply in metastatic lymph nodes is more difficult for angiogenesis compared to the primary lesion, making them more prone to necrosis during growth. However, this hypothesis need further studies to explore. However, it also can not be excluded the influence of too low number of included patients, and further a large corhort study will need to be initiated.

Furthermore, this study demonstrated the diagnostic significance of ED and ECV_V_ in evaluating which SDCT multi-parameters can predict LNM in EC. The ED value for lesions in the non-LNM group was slightly lower than in the LNM group (104.200 compared to 104.450), a novel finding that could provide new insights into predicting LNM in EC. In contrast, Nagano et al. investigated mediastinal LNM in NSCLC and found that the ED value of metastatic lymph nodes was significantly lower than that of non-metastatic ones [32]. Our investigation differs from Nagano et al.‘s work in several ways. First, the study populations were different; our research focused on EC patients, whereas previous studies targeted NSCLC. Second, our regions of interest were the primary lesions, not the lymph nodes. The extracellular matrix (ECM), a macromolecule, is integral to regulating cellular physiological processes and maintaining cell structure, thereby constituting a vital component of the tumor microenvironment. The quantification of the ECM is indicated by the ECV, which can reflect the extent of fibrosis, tumor angiogenesis, and the characteristics of the tumor’s surrounding environment. The iodine density map, generated through the post-processing of SBI data from SDCT, provides a direct visualization of iodine distribution. Consequently, only the iodine density image during the equilibrium phase is required to accurately reflect the contrast agent. By analyzing this distribution, fECV values can be derived, significantly reducing image mismatch and enhancing measurement accuracy. This study demonstrates that an elevated ECV value of the primary lesion in EC correlates with an increased likelihood of LNM, corroborating the findings of Zhang et al. [28].

This study also identified that 35.15% (45 out of 128) of lymph nodes (LNs) with a size of less than 10 mm were metastatic. Similarly, Luo et al. reported that approximately 32.93% (55 out of 167) of LNs smaller than 10 mm exhibited metastatic characteristics [33]. Our findings were consistent with these observations. Although there is a correlation between LN size and cancer involvement, CT has limitations in accurately distinguishing between nodes, as enlarged LNs may be inflammatory while normal-sized ones may be metastatic. Consequently, relying solely on the morphological parameter of size to assess LNM presents certain limitations. It is challenging to determine whether a lymph node is metastatic using CT in the absence of necrosis and extracapsular spread. This study integrates the morphological parameters of lymph nodes with the SDCT parameters of primary tumor lesions to develop a logistic regression prediction model. The logistic regression model exhibited a notable AUC of 0.85, signifying excellent predictive performance for LNM. This robust statistical validation underscores the clinical utility of these biomarkers and suggests their potential integration into routine clinical practice, thereby potentially transforming treatment planning and patient management in ESCC.

Our study is subject to several limitations that merit consideration. Firstly, the research relied on retrospective data from a single center. To improve the reliability of the evidence, it is essential to include a larger sample size and incorporate acquisition parameters from CT scanners across multiple institutions. Secondly, the study focused exclusively on the SDCT parameters of the primary lesion to predict LNM in esophageal cancer, without examining the predictive efficacy of LNM and its distribution across different regions. Thirdly, while radiomics and deep learning techniques have been extensively applied to prediction and classification tasks in various diseases, such as breast, lung, and melanoma cancers, further research is needed to obtain more comprehensive and valuable information that could enhance cancer diagnosis and treatment.

## Conclusions

In conclusion, our findings indicate that the quantitative parameters derived from SDCT, when integrated with clinical data, hold promise for predicting LNM in ESCC and exhibit satisfactory diagnostic performance and reproducibility.

## Data Availability

The datasets generated and analyzed during the current study are not publicly available because the dataset will be further studied to publish other works but are available from the corresponding author on reasonable request.

## Abbreviations

ESCC: Esophageal squamous cell carcinoma
CT: Computed tomography
LASSO: The least absolute shrinkage and selection operator
ROC: Receiver operating characteristic curve
DCA: Decision curve analysis
LNM: Lymph node metastasis
SDCT: Dual-layer spectral detector computed tomography
AJCC: American Joint Committee on Cancer
TNM: Tumor, Node, Metastasis
ROIs: Regions of interest
LR: Logistic Regression
VNC: The CT values of the lesions at virtual non-contrast
IC: Iodine concentration
Nct: Lymph node status
NIC: Normalized iodine concentration
Zeff: Effective atomic number
ED: Electron density
AEF: Arterial enhancement fraction
ECV: Extracellular volume
LAD: Tumor length measured by CT as long-axsis diameter
SAD: Tumor thickness measured by CT as short-axis diameter
